# B Cells Dynamic in Aging and the Implications of Nutritional Regulation

**DOI:** 10.3390/nu16040487

**Published:** 2024-02-08

**Authors:** Yifei Yu, Chenxu Lu, Weiru Yu, Yumei Lei, Siyuan Sun, Ping Liu, Feirong Bai, Yu Chen, Juan Chen

**Affiliations:** Key Laboratory of Precision Nutrition and Food Quality, Department of Nutrition and Health, China Agricultural University, Beijing 100091, China; hbyuyifei@163.com (Y.Y.);

**Keywords:** aging, B cells, immunity, autoimmune diseases, nutritional intervention

## Abstract

Aging negatively affects B cell production, resulting in a decrease in B-1 and B-2 cells and impaired antibody responses. Age-related B cell subsets contribute to inflammation. Investigating age-related alterations in the B-cell pool and developing targeted therapies are crucial for combating autoimmune diseases in the elderly. Additionally, optimal nutrition, including carbohydrates, amino acids, vitamins, and especially lipids, play a vital role in supporting immune function and mitigating the age-related decline in B cell activity. Research on the influence of lipids on B cells shows promise for improving autoimmune diseases. Understanding the aging B-cell pool and considering nutritional interventions can inform strategies for promoting healthy aging and reducing the age-related disease burden.

## 1. Introduction

Aging is a natural and intricate process that affects all living organisms. As time passes, physiological structures undergo changes, leading to a decline in function. These changes are characterized by a gradual reduction in adaptability and resistance, commonly referred to as aging. All systems within an organism are affected by aging, and the immune system is no exception. Serving as a vital defense mechanism, the immune system undergoes a diminishment of its capacity to respond effectively to pathogens and cancer cells as age progresses—an occurrence known as immunosenescence [1]. The outcomes of immune system aging encompass an increased susceptibility to autoimmune diseases. Fundamentally, the state of immune aging is shaped by adaptive changes in the immune system throughout the aging journey [2]. As we delve into the intricacies of adaptive immunity, it becomes evident that B cells play a central and pivotal role in orchestrating the body’s immunologic mechanisms. B cells are of paramount importance in the realm of adaptive immunity. The dysfunction in B-cell response among the elderly contributes to heightened production of autoantibodies and an increased risk of autoimmune diseases and other chronic inflammatory conditions [3]. The interplay between aging, B cell dynamics, and nutritional status intricately intertwines, forming a nexus wherein the modulation of B cell functions is closely interlinked with the nutritional milieu, thereby influencing the aging processes. Nutrients, including carbohydrates, amino acids, vitamins, and lipids, play crucial roles in regulating the immune system. Investigating how nutrition can be strategically employed to postpone pathological changes induced by aging and decrease the incidence of age-related diseases by modulating B cells remains a primary focus of research in the life sciences.

This review centers on examining how aging conditions impact both the quantity and functionality of B cells. Additionally, it delves into the contribution of B cells to chronic diseases, including systemic lupus erythematosus (SLE), primary Sjogren’s syndrome (pSS), and rheumatoid arthritis (RA). Then, we emphasized the critical role of nutritional regulation in modulating the immune process, especially the importance of lipid metabolism. Combined with the effects of lipids on B-cell immune function and autoimmune diseases, the future development of targeted drugs related to lipid metabolism will certainly be a new opportunity.

## 2. A Brief Overview of the Key Types of B Cells Involved in the Aging Process

Numerous diseases related to aging are linked to abnormalities in B cell quantity and function. Examining the role of B cells and their regulatory signaling pathways can help uncover the relationship between aging and these cells, potentially identifying therapeutic targets.

Research indicates that B-1 cells, producing IgM early in ontogeny, play a regulatory role in the development of both B-1 and B-2 cells [4]. The impact of B-1 cell antibodies extends to various diseases [5,6,7], as evidenced by a diminished B-1 cell frequency observed in individuals with relapsing-remitting multiple sclerosis when compared to those without such conditions [8]. Mature B-2 cells rely on the B-cell activating factor (BAFF)–B-cell activating factor receptor (BAFFR) signaling pathway for development and survival [9]. Additional classification involves the distinction between marginal zone (MZ) and follicular (FO) B cells, where the formation of MZ B cells occurs through the signaling pathway mediated by Notch-BRP-J [10,11]. Follicular (FO) B cells have the capacity to transform into long-lived plasma cells, generating a multitude of antibodies [12]. Regulatory B (Breg) cells, vital for immune tolerance [13], suppress the immune response by releasing immunosuppressive agents. For example, one of the Bregs subset, CD1d(hi)CD5(+), releases immunosuppressants such as interleukin-10, IL-35, and transforming growth factor (TGF-β) [14]. Despite their lower frequency among total B cells, regulatory B cells exhibit potent immunosuppressive capabilities. Age-associated B cells (ABCs), prevalent in the elderly, exhibit increased autoreactivity, elevated inflammatory factor secretion, and diminished responsiveness to external stimuli [15]. Age-related changes in the lymph nodes, such as a decrease in the number of germinal centers (GCs), a decline in the number of follicular dendritic cells and fibroblastic reticulocytes, a decrease in the production of B cells in the bone marrow, and impaired maturation of B-cell affinities, all contribute to the decline in vaccine potency with age. Senescence leads to a decrease in the number and impaired function of T follicular helper cells, which provide costimulatory, survival, and differentiation signals to B cells in the GC [16]. These cells are closely linked to the onset and progression of various diseases in the aging population, including autoimmune and chronic inflammatory conditions.

## 3. Changes in the Number and Function of B Cells during Aging

### 3.1. Aging and B-1 Cells

An elevated susceptibility to autoimmune diseases and cancer is associated with age-related impairments in the immune system. Limited information is accessible regarding modifications in human B-1 cell populations in connection with aging. Concerning cell quantities, the majority of B-1 cells are generated during fetal or neonatal stages, persisting through self-replenishment in adulthood [17]. As per Alter-Wolf’s investigation, the quantity of B-1 progenitor cells in mice remains unaffected by aging [18]. Nonetheless, the proportion of B-1 cells within the overall B cell population experiences a notable decline with age, especially advancing noticeably after reaching the age of 50 [2].

Functional analysis reveals a decline in the number of antibody-secreting cells within the B-1 cell population of elderly donors when compared to their younger counterparts [2]. While the mechanism behind the autonomous immunoglobulin secretion by B-1 cells remains only partially comprehended, certain transcription factors have been demonstrated to play a role in the secretion process in both murine and human B-1 cells [19,20]. Savage observed a noteworthy decline in the expression levels of B lymphocyte inducer of maturation program 1 (Blimp-1) and Xbox binding protein-1 (XBP-1), key promoters of immunoglobulin secretion, within the B-1 cell population isolated from aged donors, as opposed to B-1 cells sourced from younger donors. Additionally, as age progressed, B-1 cells exhibited elevated levels of PAX-5, a suppressor of the immunoglobulin secretory phenotype [2]. It is essential to highlight that this difference in the expression levels of transcription factors may bear implications for the overall immune response in aging individuals. The intricate balance of these factors seems to shift with age, potentially influencing the efficacy of the immune system in combating infections and diseases.

A noteworthy shift in the immune system of aging mice is evident in the immunoglobulin repertoire of B-1 cells [21]. CD5^+^ B cells, also referred to as B-1a cells, were initially identified within mice. These B-1a cells were found to be enduring, self-sustaining entities primarily located in the pleural and peritoneal cavities. Here, they actively synthesize natural polyreactive IgM antibodies with a skewed preference towards autoreactivity. Moreover, their Igh VH gene rearrangements notably favor the VH12 segment, resulting in antibodies that specifically target phosphatidylcholine (PtC), a prominent lipid constituent within the mucosal barrier of the gastrointestinal tract and various bacterial membranes. Consequently, the receptor repertoire of B-1a cells is predisposed towards recognizing bacterial and self-antigens, a crucial feature for swift immune responses against infections and the clearance of apoptotic cells [22]. Specifically, the gradual increase with age in VH-11-encoded PtC-bound IgH sequences within the B-1a IgH library before immunization [21]. Simultaneously, there is an observable decrease in the proportion of secreted IgM, indicating a diminishing antibody diversity. Another crucial alteration involves modifications in choline phosphate and pneumococcal capsule polysaccharides, antigens situated on the cell wall of Streptococcus pneumoniae [23]. Pneumococcal infections, notably more prevalent in older individuals [24], are intricately linked to these bacteria. In the absence of B-1a cells, animals lack the ability to resist bacterial infections due to the deficiency of natural IgM, particularly anticholine phosphate and anti-pneumococcal capsule polysaccharide (PPS)-3 [25]. This underscores the pivotal role played by B-1a cells in safeguarding against pneumococcal infections. However, research suggests a diminishing protective effect of natural serum IgM against pneumococcal infection in aged mice [26]. Moreover, B-1 cells from older mice exhibit a higher occurrence of N-additions in the antibodies they produce compared to those from younger animals. This phenomenon holds the potential to contribute to enhanced protection in older populations [27]. In summary, by observing and analyzing the results of these experiments on mice, we speculate that the decline in both the proportion and functionality of B-1 cells in older adults may compromise their ability to effectively combat microbial infections and age-related diseases, such as atherosclerosis. Despite advancements, comprehensive studies on the alterations in B-1 cells during normal aging in humans are still insufficient, leaving room for further exploration and understanding.

### 3.2. Aging and B-2 Cells

Since Hu’s 1993 report on the reduction of antibody response in aging mouse B-2 cells to T-cell-dependent antigens, extensive research has delved into the effects of aging on B-2 subpopulations [28]. Frasca’s research reveals compromised isotypic conversion in aging B-2 cells. This deficiency is linked to reduced stability of E47 mRNA and diminished AID transcription [29]. While the overall count of peripheral B-2 cells remains constant in mice, there is a subtle decline in both the quantity and proportion of FO B cell subpopulations as they age [30]. The decline in the FO B cell subpopulation may contribute to the reduction in the number of initial and memory B-2 cells in the elderly [31]. In addition to the FO lineages, aging can also impact the MZ B cell subpopulation. Numerous studies have demonstrated a highly organized morphology in the MZ region of young animals [32,33,34], with Birjandi’s study revealing a higher degree of distortion in the white pulp of old mice compared to young mice [35]. The MZ region in many older mice appears diffuse and challenging to distinguish. Frasca reported a reduced frequency of MZ B cells in old mice, with 65% of them lower than all young mice, and some showing extremely low spleen MZ B cells [36]. Similarly, Cortegano observed a decrease in MZB cells in older mice [37]. However, opposing views exist, with Allman and Miller predicting a continuous increase in age-related MZ B cells [38]. Additionally, certain conditions in older mice, such as low levels of DNA-binding proteins and IL-7, favor MZ B cell development over FO B cell development [39,40]. One plausible hypothesis proposes a transition towards increased differentiation of MZ B cells, potentially at the cost of producing follicular B cells, as mice age. Despite a potential elevated production of MZ B cells in older mice, the disruption of microstructural integrity in the MZ region might impede the efficient return of newly formed MZ B cells, resulting in a perceived decrease in the frequency of MZ B cells. Beyond the decrease in MZ B cell numbers, Turner also noted defects in antigen capture and antibody production in aging MZ B cells, resulting in a weakened T-cell-independent immune response [41]. Collectively, these studies underscore the impact of aging on B-2 cell function in both humans and mice.

### 3.3. Aging and Regulatory B Cells

Regulatory B cells (Bregs) constitute a subset of B cells characterized by their immunosuppressive roles in various immunopathological processes. These cells release a plethora of inhibitory cytokines, including IL-10 and IL-35. In different disease models, Bregs have different characteristic phenotypes. For example, Gray observed that apoptotic cells could induce CD21^+^CD23^−^ MZ B cells to produce IL-10 [42]. In addition, Kurosaki reported that in the draining lymph nodes of mice with experimental autoimmune encephalomyelitis (EAE), IL-10-producing B cells mainly showed CD19^+^CD138^+^ plasmid phenotype and played an important regulatory role in the development of EAE [43]. Within the experimental autoimmune hepatitis (AIH) model in mice, Chu noted an amplification of CD11b+ regulatory B cells, demonstrating their ability to dampen the reactivity of T helper cells [44]. However, most of the existing studies focus on the role of Bregs in human diseases, and there are few age-related studies. Given their crucial role in modulating inflammation and maintaining the equilibrium of the pro-inflammatory response from ABCs, investigating the impact of aging on both the quantity and functionality of Bregs subpopulations becomes imperative.

### 3.4. Aging and Age-Associated B Cells

Mellor and collaborators first documented a unique subset of B cells that exhibited characteristics resembling antigen-presenting cells (APCs) in 2010, displaying T-cell stimulatory properties upon activation of Toll-like receptor 9 (TLR9) [45]. This distinct B-cell subset earned the designation “age-related B cells” (ABCs) and were first identified in aging mice. Phenotypically characterized by diminished CD21 and CD11c expression alongside detectable CD95 [46,47], their classification in humans remains ambiguous due to diverse phenotypic definitions, varying marker combinations, and nomenclature complexities. While Double Negative (DN) cells are commonly considered age-associated B cells, recent reviews assert that DN cells encompass a broader spectrum than ABCs, with not all DN cell phenotypes aligning with CD21^−^, CD11c^+^, and TbeT^+^ [48,49]. As individuals age, there is a notable rise in a specific subset of B cells known for their heightened pro-inflammatory nature, termed DN B cells. These cells demonstrate an increased presence in the bloodstream of individuals afflicted with autoimmune [50,51] diseases. It is speculated that their proliferation may occur in vivo in response to either autoantigens or pathogen-derived antigens, particularly within an environment conducive to inflammation, potentially resulting in the generation of either autoimmune or protective antibodies, respectively. Despite their inability to proliferate or generate antibodies against influenza antigens, DN B cells are proficient in secreting antibodies exhibiting autoimmune reactivity [52]. The pivotal transcription factor T-bet is crucial for generating B cells exhibiting the ABC phenotype in aged mice [53,54]. Furthermore, B cell receptor (BCR) stimulation and Toll-like receptor (TLR) signaling, particularly TLR7 and TLR9, are necessary for ABCs production [55]. TLR-activated B cells, influenced by cytokines like IL-4, IL-21, and interferon-γ, facilitate the expression of T-bet [56], with a growing emphasis on exploring ABCs carrying CD11c+ and T-bet+ phenotypes [15,57,58]. The spleen witnesses a progressive rise in both number and proportion of ABCs with age, virtually undetectable in young mice, emerging at low frequency at 3–6 months, and forming a distinctive and expanding pool by 12–18 months [46]. This continuous increase persists throughout an individual’s life, potentially constituting half of all spleen B cells in mice aged 24 to 30 months [59]. In contrast to follicular (FO) B cells, ABCs are present in blood, bone marrow, and spleen regardless of age but are scarce in lymph nodes and lymphatic vessels [60]. Frasca D found that compared with young mice, ABCs in old mice have a higher metabolic profile. B cells that are highly inflammatory and secrete autoimmune antibodies are pathogenic and can also induce highly inflammatory pathogenic T cells, which has been demonstrated in both mice [61] and humans [62]. By assessing the metabolic profiles of B cells in old and young mice, the results showed that B cells in old mice showed higher mitochondrial function than B cells in young mice. These results suggest that ABCs are highly metabolic compared to FO cells, possibly because ABCs require more energy to support their secretory phenotype. In addition, ABCs of old mice showed higher mRNA expression of metabolic pathway-related enzymes than those of young mice [63]. ABCs are capable of secreting the pro-inflammatory cytokine TNF-α. Studies indicate a decline in typical B2-B cells and recirculating B cells in the spleen and bone marrow of aged mice, while ABCs show an increase [59]. These findings suggest that ABCs may contribute to the depletion of pre-B cells in older mice, impacting various stages of the B lymphoid system [64]. But this contradicts previous research, which showed that the total number of B2-B cells remained constant during aging [30]. We therefore suspect that there may be a homeostasis in which the role of B cells prior to deletion of ABCs may be modulated by other mechanisms or feedback from cell subsets to maintain the overall number of B2-B cells. In short, we still need to further study the dynamic regulatory mechanism of the B cell population. Riley’s investigation hints at ABCs potentially inhibiting multiple stages of B-cell development in the older bone marrow, with reduced RAG-2 KO receptors noted in aged mouse ABCs [55]. Crucially, a collaborative interplay between ABCs and other cells, such as natural killer (NK) cells and macrophages, may generate a pro-inflammatory microenvironment in the bone marrow of older mice. As an illustration, there is approximately a threefold expansion of natural killer (NK) cells in the bone marrow of aged mice. These NK cells have the potential to undergo activation and subsequently release TNF-α [65]. Additionally, macrophages can be prompted to generate a diverse array of pro-inflammatory cytokines, among which TNF-α is included. While the role of ABCs in immune senescence remains inconclusive, their potential involvement in shaping the aged immune landscape warrants further exploration.

## 4. The Role of B Cells in Autoimmune Diseases

Autoimmune diseases arise from a breakdown in immune tolerance, a consequence of immune disorders. In recent decades, thorough investigations have highlighted the significant involvement of both innate and adaptive immune cells, with a specific emphasis on B cells, in the initiation and progression of autoimmune disorders [66,67]. Patients with diverse autoimmune conditions, such as systemic lupus erythematosus (SLE), rheumatoid arthritis (RA), and primary Sjögren’s syndrome (pSS), consistently exhibit autoantibodies and heightened B cell activation. This underscores the crucial involvement of B cells in the development of autoimmune diseases [68,69]. Recognizing the essential role of B cell dysregulation, it is firmly established that this dysregulation is pivotal in both triggering and sustaining diseases. Changes in B cell subsets, including Bregs, memory B cells, ABCs, and others, often coincide with the onset of autoimmune diseases, as indicated by various studies. Nevertheless, the exact mechanisms and outcomes of impaired B-cell tolerance in the progression of autoimmune diseases are not fully comprehended. Ongoing research is essential to unravel the intricate interplay of immune cells and identify potential therapeutic targets for mitigating autoimmune disorders.

### 4.1. Multiple Functions of B Cells in the Pathogenesis of Systemic Lupus Erythematosus

The immune system’s function involves recognizing external pathogens and initiating a protective immune response while maintaining immune tolerance to host antigens [70]. Yet, when immune tolerance falters, the immune system directs its response towards autoantigens, leading to the onset of autoimmune diseases. SLE stands out as a severe autoimmune condition wherein autoreactive B cells play a central role in the progression of the disease and subsequent organ damage [71]. Despite the unknown origin of SLE, recent research has pinpointed various pathogenic and regulatory B-cell subpopulations linked to the pathogenesis of SLE.

#### 4.1.1. ABCs in SLE

A recently identified group of B cells referred to as ABCs, characterized by CD11c surface expression and T-bet transcription factor activity, exhibits a propensity for secreting numerous autoantibodies during systemic autoimmune responses, infections, and aging [53,54,72]. In response to stimuli like anti-CD40, IL-21, and IFN-γ, mature follicular B cells undergo differentiation into CD11c^+^T-bet^+^ ABCs [68,73]. Notably, the significant buildup of ABCs expedites the progression of SLE by producing autoantibodies and presenting autoantigens [54]. It is important to emphasize that the association with SLE is more closely linked to immune-senescence than age specifically, as SLE predominantly affects women between 20–39 years.

#### 4.1.2. Innate-like B Cells in SLE

B-1 cells and MZ B cells have the capacity to produce cross-reactive and autoreactive B cell receptors that recognize epitopes on apoptotic cells. Under typical conditions, B-1 cells and marginal zone B cells release IgM and modulate the IL-10 to maintain the tolerance of the immune system [74]. However, in systemic lupus erythematosus, activated and rapidly proliferating autoreactive B-1 and MZ B cells generate a substantial quantity of IgG autoantibodies and pro-inflammatory cytokine [75,76].

#### 4.1.3. Bregs in SLE

Beyond the production of autoantibodies, B cells play diverse roles in the immune system, encompassing functions such as antigen presentation and the secretion of cytokines [77]. A growing body of research suggests the participation of regulatory B cells in preserving immune homeostasis [78]. Throughout SLE development, B cells exhibit heightened Toll-Like Receptor (TLR) expression, including TLR2, TLR7, TLR8, and TLR9 [79]. TLR2 ligands (Pam3CSK4 and FSL1) [80], TLR4 ligands (LPS) [81], TLR7 ligands (imiquimod) [80], and TLR9 ligands (CpG-DNA) [82] have been reported to significantly induce the production of IL-10-secreting Bregs. Bregs exert immunosuppressive functions through the generation of inhibitory cytokines like IL-10 and IL-35 [83,84]. Despite this, the regulatory function of Bregs in SLE pathogenesis can be impeded by B-cell activation, co-stimulation, and alterations in cytokine signaling pathways. Dysfunction in Bregs has been noted in the course of SLE progression. Research indicates that the combination of compromised Bregs function and the expansion of autoreactive B cell subsets contributes to the disruption of immune tolerance and the initiation of autoimmune responses [85]. Despite identifying various genetic predispositions and environmental influences in SLE pathogenesis, the production of autoreactive B cells and their underlying molecular mechanisms remain largely unknown.

#### 4.1.4. Advancements in SLE Treatment of B Cells

**Figure 1:**
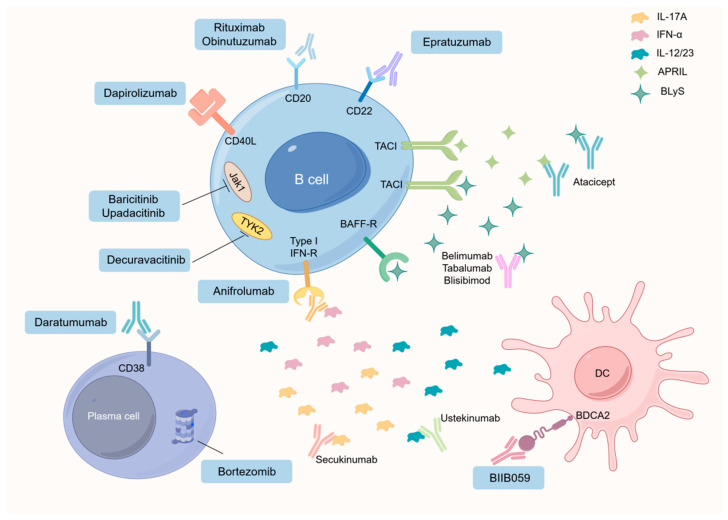
Explanation of the primary mechanism of action of compounds employed in the treatment of SLE. BAFF-R, B cell activating factor receptor; DC, dendritic cell; IL, interleukin; JAK1, Janus kinase 1; TACI, transmembrane activator and calcium modulator and cyclophilin ligand (CAML) interactor; TYK2, tyrosine kinase 2; CD, Cluster of Differentiation; IFN-R, Interferon receptor; BDCA2, blood dendritic cell antigen 2. By Figdraw.

Over the past few decades, various therapeutic strategies for treating SLE have arisen, with consideration for their impact in the context of aging. Traditional immunosuppressive drugs like cyclophosphamide aim at inhibiting proliferating activated B cells, whereas rituximab and epazumab prove effective in eliminating B cells in both human and murine lupus [86,87]. Considerable evidence advocates for suppressing cytokines like BCMA, BAFF, IL-6, and IL-21 to inhibit B cell expansion in murine lupus, but it remains essential to explore how these interventions may influence the aging-related dynamics of B cell populations [88]. Belimumab, a neutralizing antibody against human BAFF, has recently been approved. The purpose of belimumab is to inhibit the binding of soluble circulating BLyS on B cells to their target receptors (BR3, TACI, and BCMA). The ultimate goal is to block key survival signals early in B cell development and reduce the survival of autoreactive B cells [89]. Atacicept is a soluble recombinant fusion protein of the humanized Fc portion of IgG and the TACI receptor. Atacicept binds to and neutralizes BLyS and APRIL, thereby inhibiting their effects on B cells [90]. Epratuzumab is a fully humanized anti-CD22 monoclonal antibody that regulates B-cell function, leading to B-cell apoptosis [91]. Proteasome inhibition (Bortezomib) hinders the activation of the antiapoptotic nuclear factor kappa B (NF-κB) and results in the accumulation of misfolded proteins within the endoplasmic reticulum. This accumulation, in turn, triggers the terminal unfolded protein response, ultimately leading to apoptosis [92]. Delving deeper into the homeostatic control and functional interplay among various B cell and T cell populations, especially in the context of aging, holds the potential for novel perspectives on successful SLE treatment. Recognized targets within autoreactive B cells, like CD11c and T-bet, may pave the way for the creation of precise therapies for challenging cases of SLE accompanied by organ damage. Unraveling the immunosuppressive functions of Bregs may might provide opportunities for immunotherapeutic interventions targeting BREGs in individuals with SLE, including those in the aging population [93]. (Figure 1) Investigating the impaired inhibition of Bregs in the context of aging could potentially lead to the application of Bregs in SLE treatment. Despite these advancements, the functional differentiation of B-cell subpopulations in an inflammatory environment during SLE development, particularly in the aging population, remains unclear. Gaining a more profound comprehension of the inherent adaptability of B cells in vivo is essential to enhance the clinical implementation of therapies targeting B cells. Subsequent investigations should strive to unravel the mechanisms underlying the imbalance between Breg subsets and autoreactive B cells in SLE, with consideration for age-related factors. This involves unveiling distinctive functional characteristics of different B cell subsets and identifying novel targets for treating SLE. In summary, an enhanced understanding of these cell populations in SLE, taking into account the influence of aging, holds promise for advancing the clinical applicability of innovative biomarkers and validating therapeutic targets.

### 4.2. Multiple Functions of B Cells in the Pathogenesis of Primary Sjögren’s Syndrome

pSS, a prevalent autoimmune condition, primarily targets the lacrimal and salivary glands, giving rise to symptoms such as ocular dryness and xerostomia. Yet, a substantial percentage (30–40%) of individuals with pSS present varied manifestations, affecting organs like the heart, lungs, and nervous system and lymphoproliferative diseases [94]. Growing evidence underscores the prominent involvement of B cells in the pathogenesis of pSS [95]. Studies propose varied roles for B cells, either pathogenic or protective, in pSS development (Table 1). Genome-wide association studies (GWASs) have identified susceptibility genes associated with B cell activation, such as BLK, CXCR5, and PRDM1 [96]. In addition, there is an overlap between genes displaying distinct methylation patterns and those identified as risk factors in GWAS, such as BLK and CXCR5. This points to a combined impact of genetic and epigenetic dysregulation on the progression of pSS [97]. Reduced methylation of interferon regulatory genes in B cells correlates with elevated B cell counts. Changes in methylation levels within these genes in B cells show a positive association with disease activity, emphasizing the pivotal involvement of B cells in the pathogenesis of pSS [97,98].

#### 4.2.1. Bregs in pSS

The prevailing perspective suggests that the development of diverse autoantibodies, such as anti-nuclear antibody (ANA), and anti-Sjögren’s syndrome type B (anti-SSB) antibodies, are driven by autoreactive B cells and plasma cells, contributing to the advancement of pSS [111,112]. Numerous studies have unveiled additional roles of B cells in the pathogenesis of pSS, encompassing cytokine production [113] and antigen presentation [114]. A growing body of evidence underscores the diverse functional characteristics present in B cell subsets, influencing both immune responses and the development of autoimmune disorders [115]. Different Bregs’ phenotypes have been observed in connection with the onset of pSS [116,117]. Through the secretion of a variety of regulatory cytokines and effector molecules such as IL-10, IL-35, and Granzyme B (GrB), Bregs perform regulatory functions. Szabo’s identification of regulatory B cells producing IL-10, referred to as B10^+^ cells, provides valuable insights into these regulatory mechanisms [118]. In a study by Julie [119], observations revealed that the prevalence of B10^+^ cells remained stable in pSS patients when compared to healthy controls. Furthermore, B10^+^ cells facilitated Treg differentiation without influencing Th1 or TNF-α T cell differentiation. Significantly, B10^+^ cells from individuals with pSS displayed a similar capability to promote Treg responses as their counterparts in the healthy control group. Blair [99] demonstrated that CD24^hi^CD38^hi^ B cells in pSS patients reduce the frequency of Th1 and TNF-α T cells. A recent study by Lin [100] showed that CD24^hi^CD38^hi^ B cells from individuals with pSS unable to effectively inhibit the differentiation of follicular T helper cells. However, research on Breg cell function in pSS has primarily centered on CD24^hi^CD38^hi^ B cells, constituting only a phenotypically defined subpopulation. Therefore, there is much to unravel about the broader functions of Bregs in pSS.

#### 4.2.2. CD11c^+^ ABCs in pSS

CD11c^+^ ABCs, distinguished by phenotypic markers like CD11b^+^, CD11c^+^, CD21^−/low^, and CD23^low^ [46,47], undergo significant amplification in individuals with autoimmune diseases. The absence of CD11c^+^ ABCs markedly diminishes the levels of autoantibodies and disease manifestations in lupus mice, conclusively affirming the pathogenic role of CD11c+ ABCs in lupus development [47]. Recent research has pointed to a connection between the increased activity of IL-21 signaling of pSS patients, the augmentation of B cells, and heightened disease activity [120]. An increasing body of evidence supports the notion that IL-21 plays a central role in influencing CD11c^+^ ABCs in lupus patients, potentially enhancing the involvement of CD11c^+^ ABCs in the pathogenesis of pSS.

#### 4.2.3. Marginal Zone B Cells in pSS

Support for the involvement of MZ B cells in pSS comes from pSS mouse models, such as NOD-Aec1Aec2 mice [121] and Txlna transgenic mice [112]. In these models, the depletion of MZ B cells leads to the alleviation of pSS disease manifestations. Similarly, Daridon’s study [122] observed an augmentation of MZ B cells in individuals with pSS, accumulating and contributing to gland destruction through the production of autoantibodies. Nevertheless, an analysis of B cells obtained from individuals with pSS showed a significant presence of IgM^+^ CD27^+^ memory B cells in the glands. The significance of the MZ B cell subpopulation in pSS is emphasized by the observation that the majority of B-cell lymphomas associated with pSS originate from MALT (MZ B cells).

#### 4.2.4. Memory B Cells in pSS

Recent studies underscore the participation of memory B cells in the development of pSS. Some studies have found impaired memory B cell responses in older adults after influenza vaccination. Burton found that the formation of age-associated memory B cells was weakened in 67–86-year-old individuals, and the expansion of age-associated memory B cells was lower, indicating impaired GC function after vaccination in older adults [123]. Despite a decrease in the count of peripheral CD27^+^ memory B cells, these cells have a tendency to amass in the glands of individuals with pSS [69]. It is worth highlighting that CD27^+^ memory B cells demonstrate elevated levels of chemokine receptors CXCR4 and CXCR5, potentially aiding the infiltration of CXCL12 and CXCL13 chemokines into inflamed glands [110]. In individuals diagnosed with primary Sjögren’s syndrome (pSS), CD27^+^ memory B cells participate in the establishment of ectopic germinal-like structures within exocrine glands, indicating the involvement observed in transitional B cells.

#### 4.2.5. Advancements in pSS Treatment of B Cells

**Figure 2:**
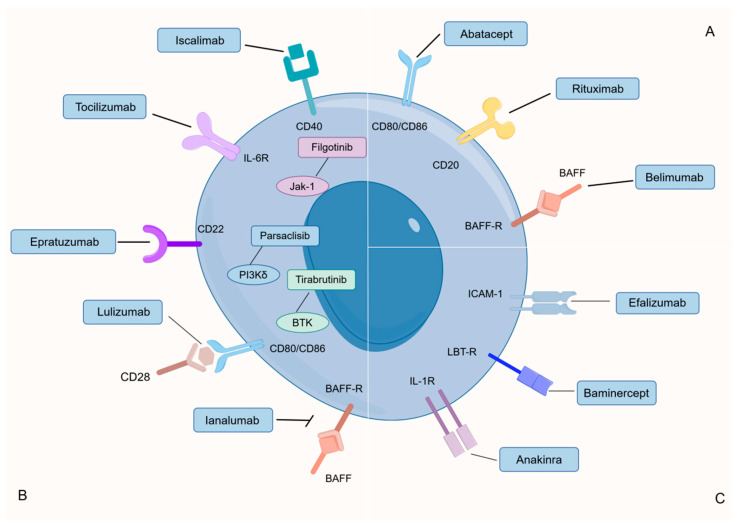
Current and potential therapies for PSS targeting B cells. (**A**) Treatments that have been approved for use in humans. (**B**) Human therapies under evaluation. (**C**) Several attempts to treat PSS have failed. BAFF: B-cell activating factor; BAFF-R: B cell activating factor receptor; LTB-R: lymphotoxin beta receptor; BTK: Bruton’s tyrosine kinase; ICAM-1: intercellular adhesion molecule 1; IL: interleukin; JAK1: Janus kinase 1; PI3Kδ: Phosphatidylinositol 3-kinase δ. By Figdraw.

Addressing pSS effectively remains a formidable challenge with no definitive cure currently known. Treatment objectives primarily revolve around symptom alleviation and the prevention of potential complications. Rituximab has emerged as a prominently studied B-cell therapy for pSS [124]. Targeting the CD20 protein on the surface of B-cells, this chimeric monoclonal antibody serves to selectively address the majority of these immune cells. The interplay between CD20 and the monoclonal antibodies is instrumental in triggering cell death, employing mechanisms like apoptosis or cellular cytotoxicity. These processes are mediated through complement-dependent or antibody-dependent pathways [125]. Gaining prominence as a potential therapeutic option for primary Sjögren’s syndrome (pSS), belimumab is a monoclonal antibody specifically designed to target the B-cell activating factor (BAFF). The presence of elevated BAFF levels in the saliva, serum, and affected tissues of pSS patients, in comparison to their healthy counterparts, has drawn attention to the exploration of belimumab’s efficacy in pSS treatment. Ongoing investigations involve Ianalumab [126], which disrupts BAFF signaling, and Epratuzumab [127], targeting CD22, a co-receptor of the B-cell receptor. Functioning as a selective co-stimulation modulator, Abatacept directly engages with CD80 and CD86, potentially diminishing the antigen-presenting capability of CD21-/low B cells [107]. Additionally, Iscalimab [128], a monoclonal antibody directed at CD40, has shown promising preliminary results. Research is actively exploring small molecules like JAK1 (filgotinib) and TYK2 (lanraplenib) for therapeutic targeting. Additionally, investigations into small molecules involved in BCR signaling, such as PI3Kδ and BTK, as potential therapeutic targets, are underway [129]. Recent findings suggest that heightened BTK activity in B cells within the peripheral blood contributes to the activation of the IL-21-mediated signaling pathway. This occurs through the induction of elevated nuclear phosphorylated STAT1 levels, particularly in individuals with autoimmune disorders [130,131] (Figure 2).

### 4.3. Multiple Functions of B Cells in the Pathogenesis of Rheumatoid Arthritis

Rheumatoid arthritis (RA) manifests as synovial inflammation, representing a fundamental pathological alteration. Characterized by symmetrical, persistent, and progressive polyarthritis, RA primarily affects small joints, with potential involvement of vital organs like the heart, lungs, kidneys, and nervous system in certain cases [132]. The pathogenesis of RA has not yet been clarified. Current studies believe that RA is caused by environmental factors acting on individuals with genetic background and inducing immunopathological reactions, among which immunohormone disorder is the central link leading to the pathogenesis of RA [133]. Recent research has extensively explored the role of B-cell dysfunction in RA pathogenesis. The reduction in regulatory B cell proportions and the disturbance in immune tolerance are pivotal factors in RA development. A comprehensive comprehension of the interplay between B-cell dysfunction and RA pathogenesis not only contributes to a clearer understanding of the disease’s origin but also provides avenues for exploring novel strategies in RA treatment.

#### 4.3.1. Bregs in RA

In recent years, multiple investigations have underscored the significance of diminished Breg numbers in the peripheral blood of RA patients coupled with disruptions in functional hormonal equilibrium as pivotal factors contributing to immune intolerance and subsequent pathogenesis [134]. Studies by Flores Borj [135] and Daien [136] revealed a substantial reduction in both the count and proportion of CD19^+^CD24^hi^CD38^hi^ Bregs in the peripheral blood of RA patients, accompanied by impaired Breg function that hindered Treg differentiation. Additionally, Cui [137] identified a decline in CD19^+^CD5^+^CD1d^hi^ Bregs in RA patients, potentially linked to increased granzyme-B (GzmB) secretion and pathogenicity. Guo [138] observed a decrease in CD19^+^TGFβ^+^ Bregs in RA patients, suggesting potential associations with organ involvement such as pulmonary interstitial fibrosis. Salomon [139] reported diminished numbers and compromised functionality of CD19^+^CD24^hi^CD27^+^ and CD19^+^CD24^hi^CD38^hi^ Bregs in RA patients. Nevertheless, the current research is constrained by a lack of in-depth exploration into Breg phenotypes, and the role of Bregs in RA pathogenesis remains a subject of debate, necessitating further investigation.

#### 4.3.2. Age-Associated B Cells in RA

ABCs exhibit low levels in healthy individuals, increasing with age and escalating significantly in cases of chronic inflammation linked to autoimmune diseases and infections. This has been demonstrated in SLE, multiple sclerosis (MS), and pSS [57,140]. Noteworthy attention has been directed towards the role of ABCs in RA. Bao [141] utilized flow cytometry to detect an elevation in ABCs (CD19^+^CD11c^+^T-bet^+^) in peripheral blood. Individuals with high disease activity exhibited a conspicuous increase in comparison to those with moderate or low disease activity and the healthy control group (*p* = 0.0002, *p* < 0.0001, and *p* < 0.0001, respectively). Following three months of clinical intervention, good responders exhibited a significant decrease in peripheral blood ABCs (*p* = 0.0174). This indicates that ABCs can potentially reflect disease activity, presenting them as a promising biomarker for assessing RA disease activity. However, among elderly patients, a rise in ABCs was specifically observed in elderly female RA patients [47]. Although previous studies suggested an association between plasmablasts and RA disease activity [142], Bao [141] demonstrated no significant increase in plasmablast proportions with disease activity, possibly attributed to individual differences. Given that ABCs express low levels of CD27, CD38, and CD138, serving as precursors to plasmablast cells, their connection with plasma cells warrants further investigation.

#### 4.3.3. Plasma Cell in RA

It is crucial to acknowledge that not all B cells contribute to RA pathogenesis, as certain antibodies produced by plasma cells can exert preventative and protective effects against RA. Naturally occurring antibodies (NAbs) [143] and therapeutic anti-citrulline protein antibodies (tACPAs) [144] fall into this category. Higher NAbs levels in autoimmune disease patients correlate with a reduced risk of cardiovascular accidents [145], while RA patients with low NAb levels exhibit a higher frequency of cardiovascular events over five years compared to those with high IgM antiphosphocholine NAbs levels [143]. Experimental models have shown that IgM-NAbs significantly decrease joint damage clinical scores, even preventing inflammatory arthritis development [145]. These findings highlight the protective effects of specific B cell-produced antibodies against RA. The mechanisms underlying NAbs’ inhibition of RA pathogenesis remain undiscovered and warrant further investigation. tACPAs, another protective antibody binding specifically to citrulline at position 3 (Cit3) of histones 2A (citH2A) and 4 (citH4), are rare and present in extremely low numbers [146]. Chirivi’s study demonstrated that tACPAs can reduce neutrophil extrinsic trap (NET) release, potentially initiating NET uptake by macrophages in vivo. This mechanism reduces joint damage and disease progression in CIA mice, suggesting new avenues for RA treatment.

#### 4.3.4. Advancements in RA Treatment of B Cells

**Figure 3:**
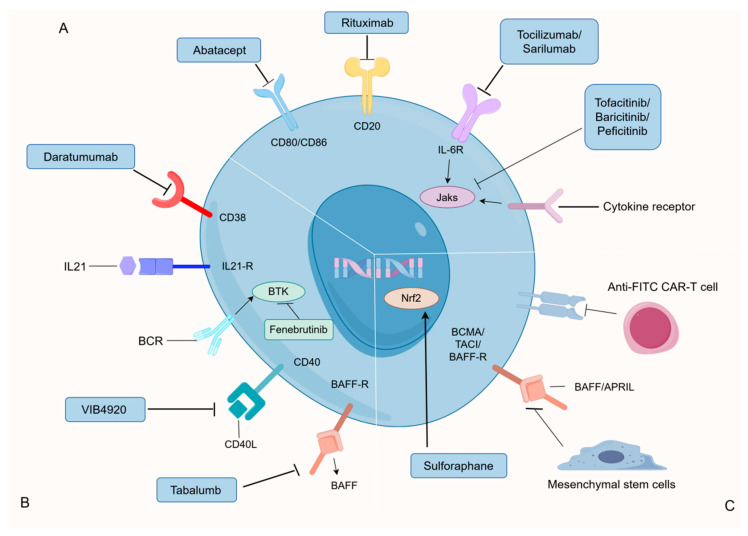
Current and potential therapies for RA targeting B cells. (**A**) Treatments that have been approved for use in humans. (**B**) Human therapies under evaluation. (**C**) Potential treatments (only tested in CIA mouse models or in vitro experiments). Jaks, Janus kinase; BTK, Bruton tyrosine kinase; BCMA, B cell maturation antigen; TACI, transmembrane activator and CAML interactor; Anti-fitc CAR T cells, anti-fluorescein isothiocyanate chimeric antigen receptor T cells. By Figdraw.

Currently, Rituximab therapy stands out as the most extensively employed B-cell targeted approach for managing RA. Positive clinical responses, including diminished synovial B cells, plasma cells, and IgG, have been observed in RA patients subjected to Rituximab treatment [147]. Rituximab is a chimeric monoclonal antibody against human CD20 that is thought to work through complement-mediated and antibody-dependent cell-mediated cytotoxicity, induction of apoptosis, and inhibition of cell growth. The key target, CD38, mainly expressed in plasma cells, is effectively addressed by Daratumumab, an anti-CD38 monoclonal antibody. In vitro experiments have demonstrated the dose-dependent removal of plasma cells and plasmablasts from peripheral blood mononuclear cells in RA patients [148]. However, further confirmation is needed regarding the efficacy and safety of Daratumab in RA patients [149]. Abatacept (CTLA-4Ig) has gained approval for RA treatment and shown success in treating autoimmune diseases. Research findings highlight Abatacept’s effectiveness in rheumatoid arthritis (RA) treatment, showcasing its ability to modulate the expression of CD80/CD86 in peripheral blood B cells. This modulation results in a simultaneous reduction in both plasma cell counts and serum IgG antibody levels in RA patients. These promising outcomes position Abatacept as a valuable therapeutic option for addressing immune dysregulation in autoimmune conditions such as RA [107]. Numerous treatments for RA are currently in development or undergoing clinical trials, encompassing anti-FITC CAR-T cells [150], Mesenchymal stem cells (MSCs) [151], BTK inhibitor (Fenebrutinib) [152], TNF inhibitor [153], JAK inhibitors [154], and Anti-IL-6R monoclonal antibodies (Tocilizumab and Sarilumab) [155], among others. (Figure 3) The precise discrimination between pathogenic and protective B cells emerges as a pivotal focus for the precision treatment of RA. Ongoing efforts hold the potential to devise therapies focused on selectively targeting pathogenic B cells responsible for autoantibody production, paving the way for innovative treatments in the future.

## 5. Nutrients Regulate B Cells and Their Implications for Autoimmune Diseases

As our understanding of the intricate connection between the immune system and metabolism advances, there is a growing recognition of the significant role nutrients play in immune regulation. This intricate interplay has profound implications for B cell development, ultimately shaping the landscape of autoimmune diseases. Recent research has delved into the specific regulatory impacts that various nutrients exert on B cell dynamics and antibody responses. Carbohydrates, amino acids, vitamins, and lipids have been subjects of extensive investigation, representing pivotal elements that influence the intricate dance of B cell activities and the production of antibodies.

### 5.1. Effects of Energy on B Cells Dynamic Changes

Sadras developed a core theory called “metabolic gatekeeper mechanism” [156]. Multiple indicators suggest that B cells inherently face persistent energy limitations owing to the unique morphological, transcriptional, and biochemical characteristics inherent in the B cell lineage [157]. Conversely, autoreactive B cells trigger notably intense and prolonged BCR signals, depleting metabolic reservoirs and leading to ATP deficiency, acute energy stress, and subsequent negative selection. The implication is that the intrinsic metabolic limitations within B cells function as a safeguard, targeting autoreactive cells by exposing them to metabolic strain triggered by hyperactivation. This strain encompasses factors such as ATP deprivation and oxidative damage, ultimately playing a role in eliminating abnormal cells. Building on the theory of metabolic gatekeepers, Sadras predicted that overnutrition would disrupt the function of B-cell gatekeepers, and it did. Numerous epidemiological findings strongly indicate that obesity and Type 2 Diabetes Mellitus (T2DM) pose as risk factors for autoimmune diseases in patients [158,159]. Meta-analytical studies have demonstrated that individuals with obesity face an elevated susceptibility to the onset of autoimmune conditions like RA [160]. Research conducted on mouse models of autoimmunity provides additional support for this evidence, showing that high-fat and high-energy diets expedite the progression and severity of autoimmune diseases [161]. Obesity, characterized by an imbalance between energy consumption and expenditure, coupled with resistance to leptin, correlates with diminished signaling through the leptin receptor (LEPR) [162]. Importantly, leptin also plays a role in modulating B cell tolerance and is implicated in the onset of autoimmune disorders [163,164,165]. Thus, these observations suggest that the high abundance of glucose and energy supplies in the context of T2DM and obesity represents a risk factor in B cell transformation. A recent study found that dietary restriction (DR) (fully nutritionally adequate but with 60% of the food intake of normally fed mice), that is, controlled energy intake, extended the health and longevity of a variety of animals. By sequencing the BCR heavy chain variable region in the spleen, we found that DR retains diversity during aging and attenuates the increase in clonal amplification [166]. A decrease in B-cell repertoire diversity and an increase in clonal expansion were associated with higher morbidity, suggesting a potential contribution of B-cell repertoire dynamics to health during aging following restriction of energy intake.

### 5.2. Effects of Carbohydrates on B Cells Dynamic Changes

By exploring the relationship between macronutrient composition and spleen B cell proportion, Tan’s study confirmed a significant positive correlation between dietary carbohydrate intake and B cell proportion in the spleen and mesenteric lymph nodes, which was not observed with other macronutrients. Further studies confirmed that mice fed a high-carbohydrate diet had a higher proportion and absolute number of splenic limbic areas and B1 cells, resulting in higher antigen-specific IgG levels after immunization [167], while high-carbohydrate diet did not affect follicular B cells [167]. This suggests that dietary management could serve as an innovative intervention to optimize immune responses or restore B lymphocyte production after immunosuppression. Glucose, a key carbohydrate, emerges as the preferred dietary substrate for B-cell development. Its influence is exerted through glycolysis and oxidative phosphorylation, reducing apoptosis in early B cell development via mTOR activation. Notably, B regs and plasma cells are recognized to favor oxidative phosphorylation. Therefore, high carbohydrate intake can save B-cell lymphogenesis due to IL-7 signaling defects [168]. Increased carbohydrate intake may also enhance the antibody response by supporting the glucose demand of long-lived plasma cells [169]. Hence, in autoimmune conditions like MS or RA, adopting a low-carbohydrate diet may diminish B cell function, offering therapeutic potential [167]. In addition, a high-carbohydrate and low-protein diet is beneficial for metabolic health and longevity [170]. The benefits of a ketogenic diet consisting of minimal carbohydrate content have been reported in animal models and in inflammatory diseases in humans [171,172].

### 5.3. Effects of Amino Acids on B Cells Dynamic Changes

Amino acids, with a focus on glutamine, serve as essential energy substrates and contribute to the antibody production mechanism. In this context, the term antibody production mechanism refers to the intricate processes and pathways through which B cells synthesize and release antibodies in response to antigens, encompassing various stages such as B cell activation, differentiation, and antibody secretion. The significance of amino acid uptake and metabolism, particularly glutamine and leucine, in activated B cells and plasma cells (PCs) is underscored by their heightened utilization [173]. Various amino acid sensors, such as Leucine-tRNA synthetase (LRS), Sestrin-2 (SESN2), and so on, initiate a complex signaling cascade that activates mTORC1 [174,175,176]. Furthermore, glutamine metabolism and ATP production via the citric acid cycle contribute to the generation of α-ketoglutarate (αKG), providing an additional activation signal for mTORC1 [177]. Together, these metabolic pathways for amino acids play a pivotal role in influencing B cell differentiation. Conversely, protein energy malnutrition may lead to a diminished plasma cell response.

### 5.4. Effects of Vitamins on B Cells Dynamic Changes

Vitamin A, along with its derivative all-trans retinoic acid, plays a significant role in shaping B cell development, guiding the differentiation of plasma cells, and influencing antibody responses. This process involves prompting Ab production through IRF4, driven by the influence of the retinoic acid receptor [178]. Vitamin C, another essential nutrient, facilitates plasma cell differentiation and antibody response through an epigenetic pathway. This includes an increased DNA demethylation process orchestrated by Tet methylcytosine dioxygenase 2 and 3 (TET2/3), resulting in heightened BLIMP-1 expression [179]. Studies also suggest that vitamin C, present during early B cell activation, supports enhanced differentiation of germinal center B cells towards plasma cell lineages [179]. In fact, despite the enhanced primary immune response to the antigen, we also observed a decrease in memory B cell and antibody titers after the second immunization following ascorbate treatment, which may be due to the interrelationship between plasma cell and memory B cell lineage differentiation [180]. Vitamin D, yet another vital nutrient, holds promise in countering autoimmune diseases. 1,25(OH)2D3 hinders B cell proliferation and plasma cell differentiation while promoting apoptosis. It augments B memory cell production, activates Breg cells, and contributes to immunoglobulin synthesis [181]. Notably, women consuming over 400 international units of vitamin D daily have shown a reduced risk of multiple sclerosis development by up to 41% [182].

In summary, nutrients present in food, including carbohydrates, amino acids, and vitamins, play a pivotal role in shaping and regulating the functionality of B cells. Glucose, particularly, serves as the primary energy substrate for B cell development, while amino acids, notably glutamine, serve as the fundamental constituents for antibody production. Vitamins A, C, and D oversee B-cell development, antibody secretion, and immune response processes by activating specific pathways and factors. A deeper exploration of the regulatory mechanisms of these nutrients on B cells is anticipated to yield novel intervention strategies and insights for optimizing immune responses and treating autoimmune diseases.

### 5.5. Effects of Lipids on B Cells Dynamic Changes

While extensive research has focused on carbohydrates, amino acids, and vitamins, there is an increasing recognition of the crucial role played by lipids in modulating B cell functions. Lipids, serving as essential constituents of cell membranes and functioning as signaling molecules, wield influence over intracellular pathways vital for B cell development, survival, and antibody production. The intricate interplay between lipids and B cells is gaining prominence due to its potential implications for therapeutic interventions in autoimmune diseases, making lipid regulation a central theme in current immunometabolism studies. This discussion centers on the impact of lipid synthesis, uptake, and catabolism on B cell function, with a specific focus on autoimmune disorders.

#### 5.5.1. Effects of Lipid Synthesis on B Cells Function

B cells, upon activation, undergo a remarkable surge in the de novo synthesis pathway of fatty acids, a process vital for their functional dynamics. The RNA sequencing analysis of both mouse B cells and those activated by LPS/IL-4 reveals a notable elevation in the expression of key genes associated with fatty acid biosynthesis [183]. In an intriguing experiment involving mice treated with the Fasn inhibitor C75, peritoneal B1a cells exhibited reduced levels of Ki67, a marker of cellular proliferation, along with a conspicuous depletion of neutral lipid stores. This resulted in a discernible reduction in the population of peritoneal B1a cells, while no significant differences were observed in other B cell subsets in the peritoneal or splenic compartments [184]. One pivotal enzyme in this process is Stearoyl-CoA desaturase (SCD), which plays a critical role in converting saturated fatty acids into monounsaturated fatty acids (MUFA). The importance of SCD extends beyond fatty acid synthesis, as it is crucial for supporting early B cell development and facilitating germinal center formation during immune responses and influenza infections [183]. These findings collectively underscore the intricate involvement of fatty acid synthesis in shaping the functional aspects of B cells, shedding light on the complex interplay between cellular metabolism and immune responses.

#### 5.5.2. Effect of Lipid Uptake and Catabolism on B Cells Function

Lipid uptake stands out as a crucial pathway essential for meeting the nutritional and energy requirements of B cells. B cells employ receptors and binding proteins, such as CD36 and fatty acid-binding proteins (FABPs), to facilitate the uptake and transport of lipids. CD36, predominantly found in resting marginal zone (MZ) B cells, has been associated with significant impacts on immunoglobulin levels and various aspects of B cell functionality [185]. Studies using CD36-deficient mice have shown that CD36-deficient B cells are less capable of plasma cell formation and many other aspects. These deficiencies result in weakened germinal center reactions, impaired class switching, and reduced antibody production in murine models [186]. Beyond its role in lipid transport, CD36 also serves as a signaling receptor within B cells. This suggests that the influence of CD36-mediated fatty acid transport on B cell proliferation and differentiation may extend beyond its direct involvement in lipid uptake. Interestingly, in high-lipid peritoneal environments, B1a cells express elevated levels of FABP and CD36 compared to follicular (FO) B cells. This adaptation in lipid transport patterns reflects the metabolic demands of B1a cells for free fatty acids in lipid-rich environments [184]. Recent findings from Weisel’s research contribute to the understanding of B cell metabolism [187]. The research highlights that germinal center B cells rely on fatty acid oxidation, rather than glycolysis, for energy generation. This revelation opens avenues for potential therapeutic interventions aimed at modulating lipid transport patterns to regulate B cell lipid uptake and metabolism effectively. Understanding these intricate pathways holds promise for developing strategies to inhibit excessive B cell lipid uptake, offering novel approaches to modulate B cell function and immune responses.

#### 5.5.3. Potential Therapeutic Strategies for Lipids in Autoimmune Diseases

Delving into the intricate regulation of lipid metabolism within B cells provides valuable insights into the potential impact of disruptions in this pathway on autoimmune diseases. This nuanced understanding opens doors to innovative approaches for addressing autoimmune conditions by targeting specific lipid metabolic pathways.

In the context of SLE, aberrant translocation patterns of Lyn and CD45 tyrosine phosphatase to lipid rafts in B cells may contribute to prolonged B cell activation and the generation of autoantibodies through B cell receptor signaling [188]. Furthermore, recent investigations have unveiled an augmented synthesis of monounsaturated fatty acids (MUFAs) facilitated by SCD-1 and SCD-2, demonstrating a positive correlation with B cell lipid accumulation [189]. Short-chain fatty acids (SCFAs) derived from the gut microbiota exhibit the capacity to diminish B cell class switching and curb the production of autoantibodies [190]. In contrast, inadequate dietary fiber intake exacerbates the progression of lupus in NZB/WF1 mice prone to the condition [191]. The specific consequences of *n*-3 polyunsaturated fatty acid (PUFA) treatment on autoreactive B cells demand further exploration. Several studies have highlighted the therapeutic benefits of dietary supplementation with *n*-3 PUFA in patients with RA and SLE, with significant improvements in disease-related symptoms [192,193]. SLE patients show promising improvements with selective S1P1 receptor modulators such as cenerimod and amiselimod [194,195]. Treatment with Fingolimod in patients with multiple sclerosis has been linked to a significant reduction in the percentage of memory B cells [196]. Statins, conventional drugs aimed at lowering lipid levels, prove beneficial for individuals with RA and SLE who have concurrent atherosclerosis [197,198].

In conclusion, diverse lipids play a dual role as both energy sources and foundational elements in B cell development. In normal immune responses, the synthesis, uptake, and catabolism of extracellular lipids intricately coordinate B cell functions, thereby influencing autoimmune diseases. Focusing therapeutic interventions on critical regulators within B cell lipid metabolism represents essential avenues for treating autoimmune diseases. In summary, we have made a summation of nutrients affecting B cells and presented it more visually in the form of a table (Table 2).

## 6. Conclusions and Future Perspectives

This review delves into the impact of aging on B lymphocytes and explores their role in autoimmune diseases prevalent among the elderly. The aging process adversely affects B cell production in the bone marrow, resulting in a decline in both B-1 and B-2 cell numbers. Additionally, the affinity and diversity of antibodies diminish with age, leading to compromised antibody responses. Bregs, pivotal for inflammation regulation and balancing the pro-inflammatory response of ABCs, achieve this through the modulation of various cytokines. Aging prompts the expansion of age-related B cell subsets, contributing to inflammation by activating proinflammatory T cell subsets and releasing cytokines.

The aging population often grapples with various health complications, and the incidence of autoimmune diseases significantly rises in elderly individuals. Recent research has consistently highlighted the substantial involvement of B cells in the onset and progression of autoimmune diseases like SLE, pSS, and RA. Consequently, future investigations should focus on a comprehensive characterization of the aging B-cell pool, encompassing alterations in cell numbers, subpopulations, and antibody responses associated with age. This knowledge will pave the way for the development of innovative, targeted therapies specifically tailored to address B cells, providing effective strategies against autoimmune diseases prevalent in the elderly.

In the context of aging and immune response, it becomes imperative to delve into the crucial role of nutrition in sustaining the immune health of elderly individuals. A well-balanced and nutrient-rich diet stands as a linchpin in bolstering immune function and alleviating the age-associated decline in B cell activity. The intake of essential nutrients, spanning vitamins, minerals, antioxidants, and omega-3 fatty acids, becomes paramount in preserving optimal B-cell function and overall immune competence in the aging population. Lipids, recognized as a pivotal nutrient, remain relatively unexplored compared to carbohydrates and amino acids in the realm of B cell research. Despite the limited investigation into the intricate relationship between lipids and B cells, there exist noteworthy instances where lipids have demonstrated the capacity to modulate B cell function effectively, leading to improvements in autoimmune diseases. This underscores the untapped potential within lipid metabolism pathways, presenting a promising frontier for the discovery of novel targets and the development of innovative drugs in the future.

In conclusion, understanding the relationship between aging and B cells is crucial for comprehending the age-related changes in the immune system and their implications for disease susceptibility and mortality. As we navigate the ever-expanding landscape of immunology and delve deeper into the intricate world of lipid modulation in B cells, the future holds promising prospects that can revolutionize our approach to autoimmune diseases. The fusion of cutting-edge technologies with the exploration of lipid regulatory pathways is poised to unlock new dimensions in our understanding of immune system dynamics. The dawn of advanced omics technologies, artificial intelligence applications, and precision medicine techniques will empower researchers to unravel the complexities of lipid-mediated processes within B cells. This comprehensive approach paves the way for personalized therapeutic interventions that target individual immune characteristics, but also provides a beacon of hope for the challenges of promoting healthy aging and reducing the burden of age-related diseases. As we envision the future, the synergy between emerging technologies and the exploration of lipid regulatory pathways emerges as a catalyst, offering a beacon of hope for advancing our understanding and treatment of autoimmune diseases, ultimately contributing to the broader landscape of human health.

## Figures and Tables

**Table 1:** Pathogenic and protective B cell subsets in pSS.

B Cell Subset	Markers	Function	Potential Therapy
IL-10^+^ Breg	CD24, CD38, CD1d, CD5, IL-10 [99,100]	Protective	-
IL-35^+^ Breg	CD138, TACI, CXCR4, IL-35 [101,102]	Protective	-
GrB^+^ Breg	CD5, GrB [103]	Protective	-
Age-Associated B cell	CD11c, T-bet, CXCR5, CD21, CD23 [104]	Pathogenic	Belimumab [105], Iscalimab [106], Abatacept [107]
Marginal Zone B cell	CD21, CD23, IgD [108]	Pathogenic	Rituximab [109]
Memory B cell	CD27, CXCR4, CXCR5 [110]	Pathogenic	-

**Table 2:** Summary of the effects of different nutrients on B cells.

Types of Nutrients	Effects of Nutrients on the Number and Function of B Cells
Carbohydrate	➀ Glucose can induce the development and function of B cells and reduce cell apoptosis [167]. ➁ Glucose inhibits AMPK and AMPK inhibits mTORC1 and in turn induces BCL6, which influences the maturation of B cells prior to PC differentiation [199]. In addition, mTORC1 helps prepare B cells for Ab secretion [200]. ➂ Glucose inhibits GSK-3, and GSK-3 inhibits forkhead box protein O1 (FOXO1) and MYC proto-oncogene (c-MYC), thereby limiting the PC differentiation [201,202].
Amino acids	➀ Extremely high amino acid utilization in activated B cells and plasma cells. ➁ Glutamine metabolism and ATP production via the citric acid cycle contribute to the generation of αKG, providing an additional activation signal for mTORC1. ➂ Protein energy malnutrition may lead to a diminished plasma cell response.
Vitamin	➀ VA plays a significant role in shaping B cell development, guiding the differentiation of plasma cells, and influencing antibody responses. ➁ VC facilitates plasma cell differentiation and antibody response. ➂ VD hinders B cell proliferation and plasma cell differentiation while promoting apoptosis. It augments B memory cell production and activates Breg cells.
Lipid	➀ SCFAs increase fatty acid oxidation in metabolic tissues and also decrease AMPK activity, which activates mTORC1. ➁ Low doses of short-chain fatty acids enhanced class switching, whereas high doses reduced AID and Blimp1 expression, class switching, somatic hypermutation, and plasma cell differentiation responses across a broad physiological spectrum. ➂ Fatty acids further promote the release of factors such as IL-10 and TGF-β by promoting histone acetylation

## Data Availability

Not applicable.
